# Effects of indoor physical environment and psychosocial stress on work performance in a Thai academic setting

**DOI:** 10.1038/s41598-026-53266-0

**Published:** 2026-05-15

**Authors:** Watcharakorn Chuthong, Vithawat Surawattanasakul, Wuttipat Kiratipaisarl, Sasawat Navasedthakul, Ratchapong Pakdeewiwat, Sukrit Rukkhachat, Sithichode Sivaroroskul

**Affiliations:** 1 Department of Occupational and Environmental Medicine, Bankhai Hospital, Rayong, Thailand; 2 Department of Preventive and Social Medicine, Faculty of Medicine, Chulalongkorn University, Bangkok, Thailand; 3 Department of Community Medicine, Faculty of Medicine, Chiang Mai University, Chiang Mai, Thailand; 4 Environmental and Occupational Medicine Excellence Center, Faculty of Medicine, Chiang Mai University, Chiang Mai, Thailand; 5 Faculty of Medicine, Chiang Mai University, Chiang Mai, Thailand

**Keywords:** Indoor Environmental Quality, Effort-Reward Imbalance, Ventilation, Psychosocial Stress, Work Performance, Health care, Health occupations, Psychology, Psychology, Risk factors

## Abstract

While physical indoor environmental quality (IEQ) and psychosocial stress are known to affect employee well-being, their combined influence on multidimensional work performance remains underexplored, particularly within academic medical settings in Southeast Asia. To address this gap, this cross-sectional study investigated the associations between specific work performance domains and both physical and psychosocial environments among office workers in a Thai academic medical institute. A survey was conducted among 419 administrative staff members (51.16% response rate) employed for over six months. Participants completed paper-based questionnaires assessing: (1) demographics, (2) physical indoor environment perceptions (7-point Likert scale), (3) psychosocial environment (Thai Effort–Reward Imbalance Questionnaire), and (4) work performance (Individual Work Performance Questionnaire). Multiple linear regression analyzed relationships between these factors and three performance domains: task performance (TP), contextual performance (CP), and counterproductive work behavior (CWB). While TP was negatively associated with male gender (β = -0.14, 95% CI: -0.26 to -0.03), CP was significantly hindered by perceived stagnant air movement (β = -0.07, 95% CI: -0.12 to -0.02) and facilitated by adequate privacy (β = 0.06, 95% CI: 0.01 to 0.11). Furthermore, high-risk effort-reward imbalance was a strong predictor of lower CWB scores (β = -0.26, 95% CI: -0.45 to -0.07), indicating that highly stressed individuals engaged in more frequent counterproductive behaviors. These findings demonstrate that physical and psychosocial factors are significantly associated with specific performance dimensions. Specifically, poor air movement and a lack of privacy are linked to lower contextual performance, while work stress is positively correlated with counterproductive behavior. Consequently, institutional policies should prioritize improving ventilation, enhancing workplace privacy, and balancing effort-reward structures to optimize staff productivity and well-being.

**Supplementary Information:**

The online version contains supplementary material available at 10.1038/s41598-026-53266-0.

## Introduction

Indoor Environmental Quality (IEQ) plays a crucial role in workplaces, especially since most individuals spend the majority of their time indoors^1^. Office-like environments are particularly sensitive to IEQ due to the nature of the work, which often requires employees to remain indoors throughout the entire workday. Recent studies have shown that IEQ affects health, well-being, and productivity, highlighting the importance of providing a comfortable indoor environment^2^. IEQ refers to environmental factors within indoor spaces that influence occupants’ activities^3^. These factors vary across different models but generally fall into two dimensions: (1) tangible factors such as temperature, humidity, ventilation, air quality, noise, and lighting, and (2) intangible factors related to architectural design, including office layout, sense of privacy, and interior decoration^2^. Many of these elements, particularly the intangible ones, are subjective and difficult to measure directly. Therefore, questionnaires assessing occupants’ perceptions and satisfaction levels are essential for evaluating IEQ^3,4^. This approach is often referred to as Perceived Indoor Air Quality (Perceived IAQ), which reflects individuals’ subjective evaluation of physical indoor conditions^5^. In non-industrial buildings, where pollutant levels are often below detectable limits, subjective assessments can serve as valuable complements to objective measurements^4^.

The psychosocial work environment also plays a critical role in affecting workers’ health and well-being. This refers to non-physical aspects of work, such as stress and other mental or emotional demands^6–8^. The definition of the psychosocial work environment varies, but it generally refers to non-physical aspects of work, particularly psychosocial hazards and stressors. These terms are commonly used to describe negative influences of work that may pose risks to workers’ health^9^. Psychological hazards and work-related stress are often considered interchangeable, both in terms of their sources and effects, and are widely recognized as significant concerns among employees^7^. The assessment of the psychosocial work environment is typically guided by theoretical models that emphasize its relevance to specific health and organizational outcomes^7,9^. Although other methods for evaluating work environment characteristics exist, questionnaires are the most commonly used approach for assessing psychosocial factors. This is because the perception of psychosocial hazards often has a greater impact than their mere presence^9^. One widely recognized model is the Effort-Reward Imbalance (ERI) model, which conceptualizes job stress as a mismatch between high efforts expended and low rewards received. Individuals with a high ERI ratio are considered at greater risk and tend to report higher levels of job-related stress, along with negative consequences for their health and well-being^8^.

**Fig. 1:**
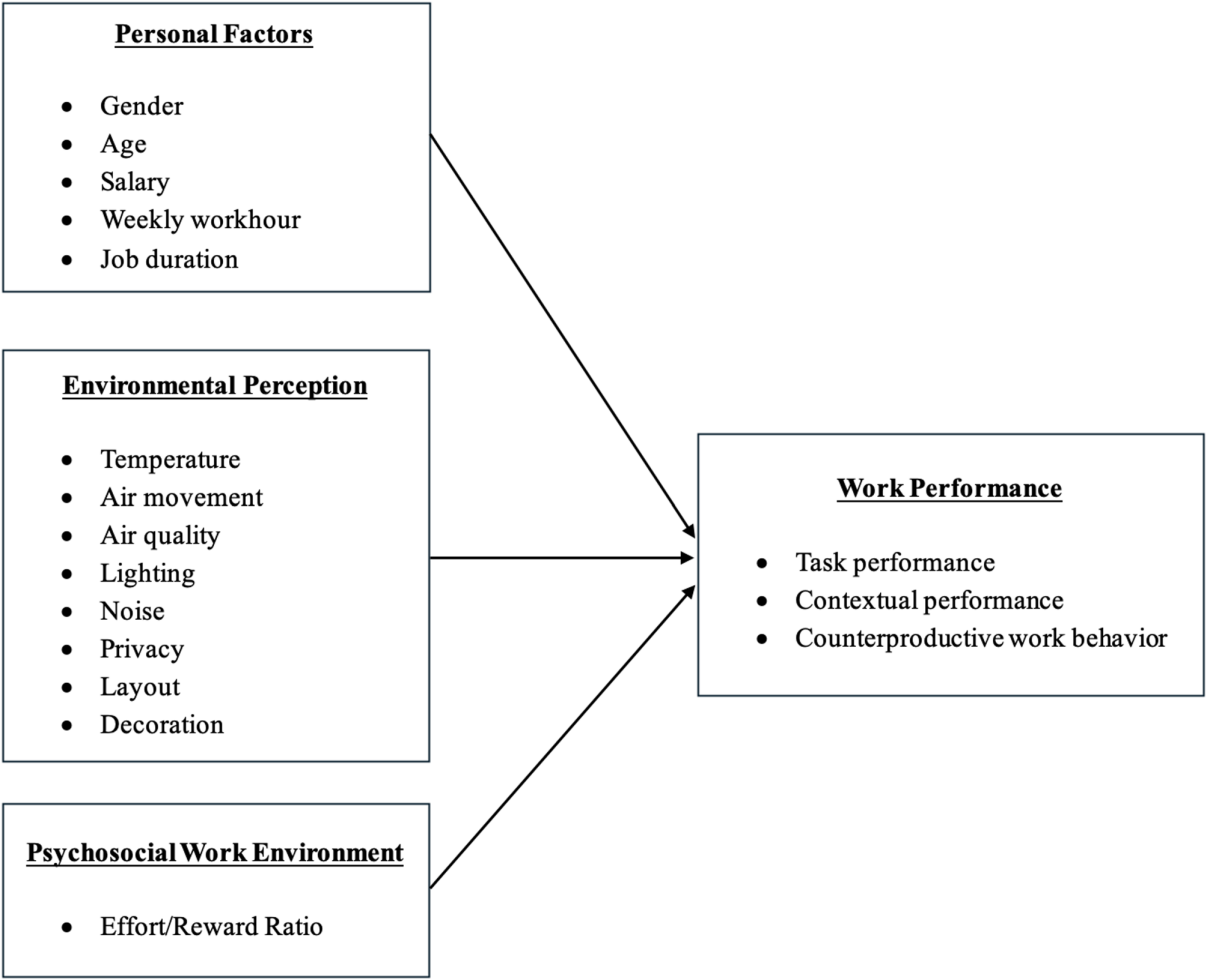
Conceptual framework of the research.

This study examined how these physical and psychosocial environment factors influenced work performance. Individual work performance (IWP) is commonly defined as employee behaviors that align with organizational goals and job responsibilities, emphasizing actions rather than outcomes (Koopmans 2013). It is typically categorized into three dimensions: task performance (TP), contextual performance (CP), and counterproductive work behavior (CWB) (Koopmans 2013;^10^. TP refers to the effectiveness with which individuals perform core duties, including the application of their technical skills. CP encompasses behaviors that contribute to the organizational and social climate, such as showing initiative, supporting colleagues, and maintaining effective communication. Contemporary organizational research highlights the importance of this dimension, demonstrating that structural inputs often translate into performance outcomes through discretionary, socially oriented behaviors, such as prosocial voice, which are broadly conceptualized as CP. Jarrar^11^,. In contrast, CWB includes actions that negatively affect the organization, such as absenteeism, distractions, theft, or substance misuse^10^. Numerous prior studies have demonstrated that IEQ can significantly influence work performance, particularly in office-based settings (Wolkoff 2021). Broader empirical evidence indicates that structural and environmental conditions shape behavioral outcomes largely through subjective perceptual mechanisms^12,13^. This strongly aligns with our theoretical framework and reinforces the necessity of examining subjective environmental perceptions rather than relying solely on objective physical metrics. Based on the existing literature, this study developed a conceptual framework to evaluate this multidimensional work environment (Fig. 1). Specifically, we hypothesized that physical environmental factors and psychosocial factors act as independent, additive predictors of individual work performance, rather than interacting synergistically.

In Thailand, IEQ has been evaluated in various buildings^14,15^. However, existing studies have primarily focused on environmental assessments without adequately examining their implications for work performance and have rarely considered the combined role of psychosocial work factors. In addition, evidence from academic medical settings remains limited. To address these gaps, this study investigates the effects of both the physical and psychosocial work environments on work performance among office workers in a Thai academic medical institute. By integrating these dimensions within a single framework, this study provides novel insights into how physical and psychosocial workplace factors jointly influence employee performance in this specific context.

## Methodology

This is a cross-sectional study which was approved by the Institutional Review Board at Faculty of Medicine, Chiang Mai University in accordance with the Declaration of Helsinki. (Reference number: 119/2023; Date of approval: March 19, 2023) Clinical trial number: not applicable.

### Study setting and population

This study collected data from a total of 819 office workers across 14 support units and 22 medical departments within an academic medical institute. Eligibility criteria required participants to have been employed at the institute for a minimum of six months and to spend at least six hours per day working in an office environment. Therefore, the final sample consisted exclusively of non-clinical administrative support personnel and did not include clinicians or direct healthcare providers. We utilized total population sampling inviting all eligible employees who met these criteria to participate. In terms of the sample size, it was calculated based on a finite population, with a minimum of 410 determined and deemed appropriate^16^. The formula was:

$$\:n=\frac{N{\sigma\:}^{2}{z}_{1-\frac{\alpha\:}{2}}^{2}}{{d}^{2}\left(N-1\right)+{\sigma\:}^{2}{z}_{1-\frac{\alpha\:}{2}}^{2}}$$

N: Population size.

σ: Standard deviation which based on newly developed questionnaire using value form Individual Work Performance Questionnaire (IWPQ) which is 0.73.

d: The accepted margin of error, set at 0.05.

α: Alpha was set at 0.05.

### Data collection

Data for this study were collected using a paper-based questionnaire in Thai, which was directly distributed to all participants through their department representatives between May and August 2023. The questionnaire comprised three parts as follows:

Part 1: General information: This section gathered data on gender, age, salary, weekly workhour, and job duration.

Part 2: Environmental perception: This section is designed to gather information on their individual office environment perception by using questionnaire which each domain developed based on the literature review that related to Thailand workplace^5^. Responses were measured on a seven-point Likert scale across eight domains: temperature, air movement, air quality, lighting, noise, privacy, layout, and decoration. Seven of these domains ranged from unsatisfactory (1) to satisfactory (7). However, the air movement scale was descriptive, ranging from draughty (1) to still (7). This variable was not reversed or centered for the analysis. Therefore, higher scores explicitly represent a perception of still air, which conceptually aligns with poorer ventilation. The tool demonstrated acceptable psychometric properties, yielding a Content Validity Index (CVI) of 0.83 and a Cronbach’s alpha of 0.72 for internal consistency.

Part 3: Effort-reward imbalance: This section is designed to gather information on psychosocial work environment by using Thai Effort-reward imbalance questionnaire (Thai ERIQ) with 23 items which had 3 domains including effort, reward and overcommitment. In this study we use data only effort and reward domain for risk-group calculation. For reward, there were 3 subdomain including self-esteem, job security, and job promotion and salary^17^. ERI ratio was calculated by using formula Ratio = E/(R*0.5454). Ratio more than 1 is high risk group which mean worker can perceive the imbalance and leads to work stress. In this study we use ERI questionnaire in Thai version which content validity and reliability were tested Content Validity Index was 0.95 and Cronbach’s alpha coefficient was 0.88^17^.

Part 4: Work performance: This section aimed to assess employees’ performance using the Individual Work Performance Questionnaire (IWPQ), which consists of three domains: task performance (5 items), contextual performance (8 items), and counterproductive work behavior (5 items). Responses were rated on a five-point Likert scale, ranging from “seldom (1)” to “always (5)” for task and contextual performance, and in reverse order from “never (5)” to “often (1)” for counterproductive behavior^18^. For each subscale, a mean score was calculated by summing the item scores and dividing by the number of items. The resulting scores range from 1 to 5, with higher scores indicating stronger task and contextual performance, or less frequent counterproductive behavior. In this study, the validated Thai version of the IWPQ was used, which demonstrated good internal consistency with a Cronbach’s alpha of 0.93^19^.

### Statistical analysis

After excluding errors and incomplete responses, the general information of a sample group was analyzed and presented using descriptive statistics, including frequency distributions and percentages. Scores from environmental perception, ERI questionnaire, and IWPQ were presented in terms of mean and standard deviation (SD). To determine the independent relationships between individual work performance and associated factors, a multiple linear regression analysis was conducted. Variable selection was entirely theory-driven to avoid omitted variable bias. All theoretically relevant covariates identified in our literature review (including personal factors, all IEQ domains, and the ERI ratio) were entered simultaneously into the full model. Standardized beta coefficients were accompanied to assist the assessment of clinical significance. Hierarchical regression was carried out separately across three blocks of variables, including the demographics variables, IEQ and ERI ratio respectively to assess the difference of coefficients and their interval estimates. Model performance in the dataset was each determined with adjusted R^2^. Prior to running the regression, these variables were assessed for multicollinearity by calculating the variance inflation factor (VIF), which should not exceed 10, and tolerance values greater than 0.1^20^. The study set a significance level at *p* < 0.05. All data analysis was performed and analyzed using Stata version 18.0 (Stata Corp., College Station, TX, USA).

## Results

### Characteristics of the study participants

A total of 419 complete responses were collected. As a result, the response rate was 51.16%. The sample consisted of 264 females (63.01%). The mean age was 38.26 (SD = 10.70). Most respondents 71.60% had monthly salary between 15,000 and 30,000 baht. Average weekly workhour and job duration were 37.83 h./wk. (SD = 8.75) and 11.47 years (SD = 10.52), respectively. Detail as shown in Table 1.

**Table 1:** Characteristics of the study participants (*n* = 419).

Variable	*N* (%)
**Gender**	
Male	155 (36.99)
Female	264 (63.01)
**Age** in year, mean (S.D.)	38.26 (10.70)
**Salary**	
< 15 K	47 (11.22)
15–30 K	300 (71.60)
30–45 K	41 (9.79)
45–60 K	10 (2.39)
> 60 K	21 (5.00)
**Job duration** in year; mean (S.D.)	11.47 (10.52)
**Weekly work hours** in hour; mean (S.D.)	37.83 (8.75)

### Environmental perception in office

**Fig. 2:**
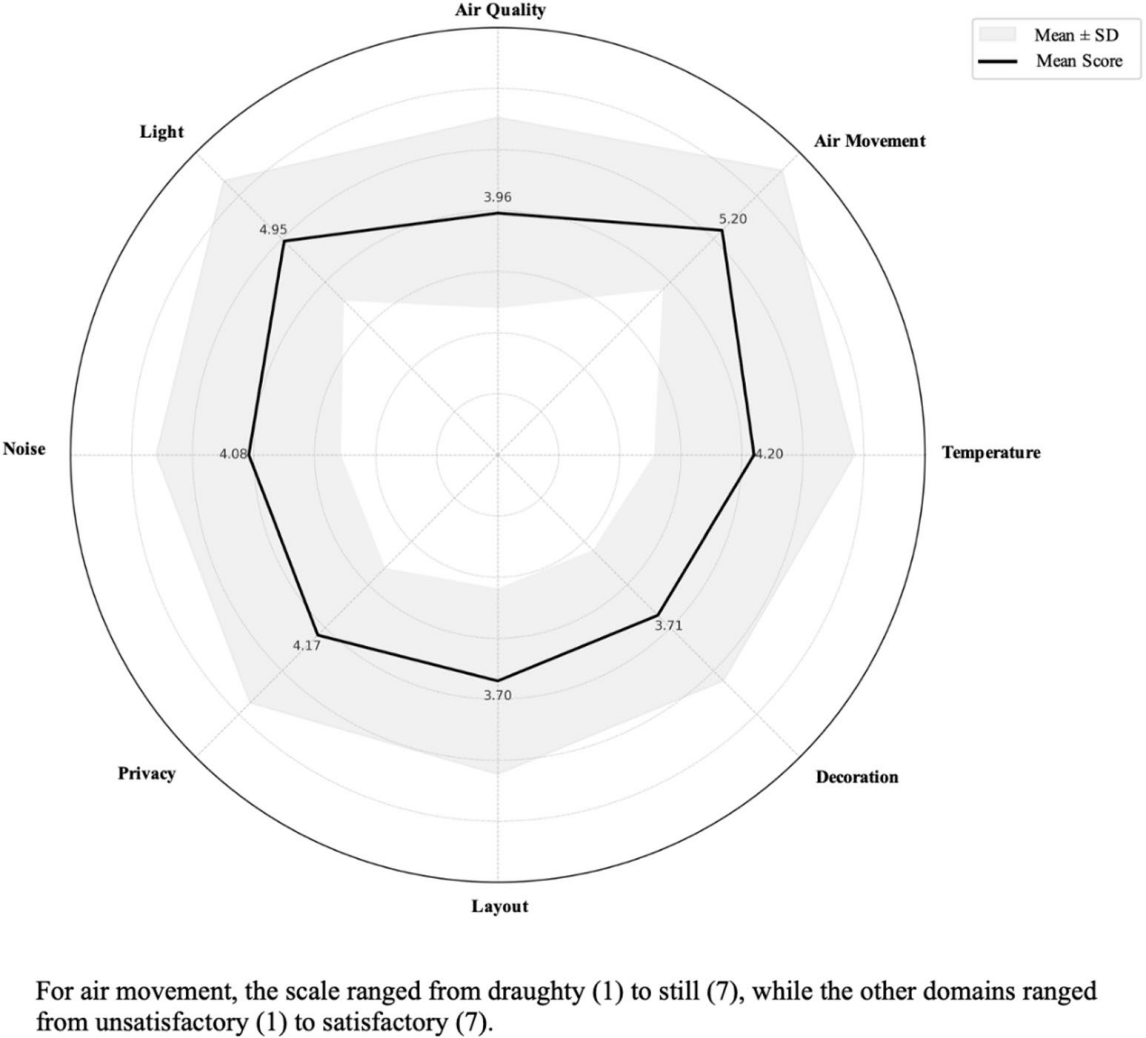
Environment perception scores (*n* = 419).

Environmental perception score was shown in Fig. 2. Regarding environmental perceptions, air movement received the highest absolute score (x̄ = 5.20, SD = 1.39). Because this scale measures physical state rather than satisfaction, this indicates that participants predominantly perceived the air in the room as relatively still and lacking circulation. Satisfaction scores for the other environmental factors ranged from 3.70 to 4.95, including temperature (x̄ = 4.20, SD = 1.65), air quality (x̄ = 3.96, SD = 1.57), lighting (x̄ = 4.95, SD = 1.40), noise (x̄ = 4.08, SD = 1.52), privacy (x̄ = 4.17, SD = 1.57), layout (x̄ = 3.70, SD = 1.53), and decoration (x̄ = 3.71, SD = 1.52). Overall, the findings suggest a moderately positive perception of the office environment, as all mean scores exceeded the midpoint of the 7-point scale.

### Effort-reward imbalance (ERI)

For psychosocial work environment, the results showed that both effort and reward mostly in high tertile (57.04 and 67.54 respectively) with average of 23.04 (SD = 4.04) and 44.14 (SD = 6.45) respectively. ERI ratio showed that there are 132 people (31.50%) in the high-risk group for work stress and 287 people (68.50%) in the low-risk group.

### Individual work performance

Work performance was evaluated across three domains. The mean score for TP was 4.27 (SD = 0.53), CP was 3.89 (SD = 0.60), and CWB was 3.80 (SD = 0.81). The researchers used the mean scores from IWPQ to examine associations with physical and psychosocial work factors. Multiple linear regression analysis identified significant relationships in each domain: TP was negatively associated with male gender (β = −0.14, 95% CI: −0.26 to −0.03); CP was negatively associated with air movement (β = −0.07, 95% CI: −0.12 to −0.02), indicating that perceptions of still air correspond to lower CP, and positively associated with privacy (β = 0.06, 95% CI: 0.01 to 0.11); CWB scores were negatively associated with being in the high-risk group for ERI (β = −0.26, 95% CI: −0.45 to −0.07), indicating that these high-risk individuals reported more frequent CWB. These results were detailed in Table 2. Multicollinearity was tested using the VIF and tolerance values. The average VIF for all variables was 2.23, with individual VIFs ranging from 1.11 to 4.89 and tolerance values were between 0.20 and 0.93. These results denoted that the independent variables were not collinearity.

**Table 2:** The associated factors with individual work performance scores by a multiple linear regression analysis (*n* = 419).

Variable	Task Performance (Adjusted R² = 0.068)					Contextual Performance (Adjusted R² = 0.020)					Counterproductive Work Behavior (Adjusted R² = 0.038)				
	Beta	95%CI		p-value	Standardized beta	Beta	95%CI		p-value	Standardized beta	Beta	95%CI		p-value	Standardized beta
		Lower	Upper				Lower	Upper				Lower	Upper		
Gender															
Female	reference	-	-	-	-	reference	-	-	-	-	reference	-	-	-	-
Male	-0.14*	**-0.26**	**-0.03**	**0.02**	**-0.13**	-0.09	-0.22	0.05	0.21	-0.06	-0.03	-0.21	0.15	0.75	-0.02
**Age**	0.01	-0.01	0.02	0.60	0.06	-0.01	-0.02	0.01	0.56	-0.03	0.01	-0.01	0.03	0.31	0.12
**Monthly salary**															
< 15000	reference	-	-	-	-	reference	-	-	-	-	reference	-	-	-	-
15001–30000	0.09	-0.17	0.19	0.92	0.01	-0.19	-0.40	0.01	0.07	-0.15	-0.08	-0.36	0.19	0.55	-0.04
30001–45000	0.05	-0.21	0.31	0.72	0.03	-0.12	-0.42	0.18	0.43	-0.07	0.04	-0.37	0.45	0.85	0.02
45001–60000	0.32	-0.09	0.74	0.13	0.09	0.12	-0.36	0.60	0.63	0.02	0.31	-0.34	0.95	0.35	0.06
> 60000	0.27	-0.07	0.61	0.12	0.11	0.13	-0.26	0.52	0.52	0.03	0.15	-0.38	0.68	0.58	0.05
**Weekly Workhour**	0.01	-0.01	0.02	0.77	0.01	-0.01	-0.02	0.01	0.10	0.04	-0.01	-0.02	0.01	0.77	-0.06
**Job Duration**	0.01	-0.01	0.02	0.49	0.08	0.01	-0.01	0.02	0.63	0.05	-0.01	-0.03	0.01	0.30	-0.12
**Office environment satisfaction**															
Temperature	-0.01	-0.05	0.04	0.84	-0.01	-0.03	-0.08	0.02	0.27	-0.08	-0.01	-0.08	0.06	0.85	-0.01
Air movement	-0.01	-0.05	0.04	0.79	-0.02	-0.07*	**-0.12**	**-0.02**	**<** **0.01**	**-0.14**	0.07	-0.01	0.13	0.06	0.10
Air Quality	0.03	-0.02	0.08	0.27	0.09	0.02	-0.04	0.08	0.50	0.05	-0.05	-0.13	0.04	0.27	-0.09
Light	0.02	-0.03	0.06	0.41	0.05	-0.01	-0.06	0.04	0.75	-0.02	0.01	-0.06	0.08	0.83	0.01
Noise	0.01	-0.03	0.05	0.65	0.03	-0.03	-0.08	0.02	0.23	-0.08	-0.04	-0.11	0.03	0.24	-0.07
Privacy	0.03	-0.02	0.08	0.28	0.08	0.06*	**0.01**	**0.11**	**0.04**	**0.15**	0.04	-0.04	0.12	0.30	0.08
Layout	0.03	-0.03	0.10	0.33	0.09	-0.05	-0.13	0.03	0.20	-0.12	-0.06	-0.16	0.05	0.28	-0.11
Decoration	-0.03	-0.10	0.03	0.34	-0.09	0.01	-0.06	0.09	0.71	0.03	0.09	-0.01	0.19	0.08	0.16
**ERI ratio**															
Low-risk (≤ 1)	reference	-	-	-	-	reference	-	-	-	-	reference	-	-	-	-
High-risk (> 1)	-0.11	-0.23	0.02	0.09	-0.09	-0.04	-0.18	0.10	0.58	-0.04	-0.26*	**-0.45**	**-0.07**	**<** **0.01**	**-0.14**

*p-value < 0.05.

## Discussion

This study examined the relationship between work performance, environmental perception, and effort-reward imbalance. It addressed both physical and psychosocial aspects of the office work environment. A new questionnaire was developed to capture employees’ perceptions of the physical indoor environment, based on a comprehensive review of the literature. Participants generally reported satisfaction with temperature, air quality, lighting, noise, privacy, layout, and decoration. However, many perceived the air movement in their workspace as relatively stagnant. Psychosocial factors were evaluated using the Thai version of the ERI scale. About one-third of workers were identified as being at high risk of psychosocial stress. Work performance was assessed using the IWPQ. High scores were observed across all three domains: TP, CP, and CWB. Multiple linear regression analysis indicated that air movement, privacy, male gender, and high-risk ERI status were significantly associated with work performance.

### Environmental perception

In this study, the mean satisfaction scores across environmental domains were moderate, indicating that employees were only slightly satisfied with most aspects of their indoor work environment. This relatively high proportion of neutral responses suggests that the current environmental conditions met basic functional expectations but failed to exceed them in a way that generates strong positive ratings. Because the environmental perception domains in this study are consistent with global IEQ frameworks, our findings suggests that even in this specific cultural and occupational setting, achieving high environmental satisfaction requires moving beyond mere compliance to actively optimizing workspace comfort settings^5,21,22^.

### Perceived air movement

A primary finding of this study demonstrated a significant negative association between perceived air movement and CP, whereas TP and CWB remained unaffected. The stability of TP suggests that suboptimal air movement does not necessarily impede the execution of core occupational tasks; rather, it depletes the psychological resources required for discretionary behaviors. Framed within ego depletion and psychosocial mediation theories, poor air movement likely functions as an environmental stressor, serving as a proxy for accumulating ambient pollutants^23^. The cognitive effort required to maintain attention under such conditions diminishes self-regulatory capacity, which is essential for prosocial and cooperative behaviors that require individuals to override self-interest^24^. Consistent with this mechanism, organizational conditions have been shown to influence discretionary behaviors through psychosocial mediators. For instance, prosocial voice has been found to mediate the relationship between safety training and safety behaviors, illustrating how contextual factors shape discretionary engagement^11^. Similarly, our findings suggest that perceived IEQ, specifically adequate air movement, acted as a contextual prerequisite that either facilitate or constrain the social engagement underlying CP. Thus, adequate ventilation is imperative not solely for baseline cognitive function^23^, but for sustaining the collaborative dynamics of the organizational environment.

### Privacy

Privacy exhibited a significant positive correlation with CP, with no significant relationship observed for TP or CWB. This finding indicates that the architectural capacity to regulate personal space is a critical antecedent to social engagement. Adequate privacy mitigates cognitive overload and facilitates stress recovery, enabling employees to regulate social interactions effectively^25^. Environments characterized by frequent interruptions and spatial intrusions, frequently observed in open-plan configurations, compromise this regulatory capacity^26,27^. Conversely, when privacy was preserved, employees retain the cognitive bandwidth necessary to engage in voluntary prosocial behaviors^26,28^. This suggests that the provision of private or semi-private spatial configurations constituted a viable structural intervention to cultivate a supportive workplace culture.

### Other IEQ factors

No significant associations were observed between performance metrics and other perceived environmental factors, including temperature, air quality, lighting, noise, layout, and decoration. Although experimental and acute-exposure studies frequently associated extremes in these parameters with cognitive and operational deficits^23,29^, the null findings in the present study might reflect longitudinal sensory and psychological adaptation. Occupants in stable office environments might develop a tolerance to moderate environmental deviations over time^30^. Furthermore, a negative association was observed between male gender and TP. Given the reliance on subjective self-report instruments, this result had to be interpreted cautiously, as it might signify a demographic reporting bias rather than an objective decrement in productivity. Additionally, the absence of stratified analyses controlling for specific occupational roles within the administrative cohort limited the definitive interpretation of this variance.

### Work stress

Analyses of work stress indicated that approximately one-third of participants met the criteria for high-risk classification. Notably, elevated ERI was significantly and negatively associated with CWB scores, demonstrating that employees experiencing occupational stress exhibited an increased propensity for counterproductive behaviors. When organizational demands necessitated high effort without commensurate rewards or support, it precipitated destructive deviance^31–33^. This imbalance may also reflect a breakdown in perceived structural fairness and organizational support, which are critical determinants of behavioral outcomes. Evidence from studies examining structural and process-related factors indicates that perceived quality and organizational conditions are closely linked to performance and engagement through subjective evaluations of fairness and support^12,13^. Consequently, interventions aimed at mitigating CWB should prioritize structural remediation of organizational equity rather than relying solely on individualized stress-management approaches. The absence of significant associations between work stress and either TP or CP suggests that, within this sample, individual coping mechanisms or the highly structured nature of administrative tasks may buffer the immediate effects of psychosocial stressors on core task execution, thereby compartmentalizing stress responses rather than impairing task proficiency^34,35^.

A major strength of this study lies in its comprehensive assessment of both physical and psychosocial aspects of the indoor work environment, offering a holistic view of their impact on work performance. The use of validated measurement tools, including the Thai version of the ERI Questionnaire and the IWPQ, enhances the reliability and cultural relevance of the findings Additionally, the relatively large sample size drawn from an academic medical institution provides robust statistical power and enhances the applicability of the findings to real-world office settings. Also, the associated factors found from the hierarchical regression analysis (Supplementary Information) was concurred to the full model (Table 2).

This study has limitations. First, this is a cross-sectional study which faces constraints in determining causal relationships between factors associated with work performance. While our findings align with established theoretical frameworks regarding indoor environments, the possibility of bidirectional relationships, such as preexisting work stress theoretically influencing how an employee perceives their physical surroundings, cannot be entirely dismissed. Second, all variables were self-reported, collected via the same questionnaire, and measured at the same time. This introduces a risk of common method variance (CMV). While statistical checks, such as Harman’s single-factor test, can be used to estimate this bias, they remain imperfect solutions. Third, because perceived IEQ is inherently subjective, individual mood, expectations, or job stress may have influenced participants’ ratings, leading to potential perception bias. Moreover, subjective IEQ measurement may contribute to high variation in data resulting in low adjusted R^2^ of the models. Fourth, the practical significance of the statistically significant finding was small according to the interpretation of standardized beta coefficients according to the Cohen’s d criteria (d < 0.2). Furthermore, the sample was drawn from a single occupational setting, which may limit the generalizability of the findings to other industries or work environments. Finally, perceived IEQ, ERI and work performance can fluctuate over time, while this study measured them at a single point. Despite these limitations, the present findings provide valuable insight into the complex interplay between employee performance with physical and psychological conditions at work.

Future research should employ longitudinal or panel designs to clarify causal relationships among perceived IEQ, effort-reward imbalance, and work performance over time. Multi-site or cross-industry samples are also recommended to improve the generalizability of findings. Integrating objective environmental measurements with subjective perceptions would help clarify the mechanisms linking IEQ and performance. Experimental interventions focusing on improving workplace design or balancing effort-reward structures are also warranted. Moreover, exploring moderating factors such as job control or organizational culture could yield a more comprehensive model of occupational performance.

## Conclusion

This study highlights how physical indoor environments and psychosocial stress shape staff performance in an academic medical institute. Perceived air movement and privacy are positively associated with CP. Furthermore, the presence of ERI in one-third of participants is correlated with lower CWB scores, indicating increased counterproductive behaviors. These findings suggest that suboptimal conditions may relate to the depletion of employees’ self-regulatory resources, which corresponds to lower social and discretionary efforts even when core tasks remain stable. Organizations are encouraged to improve indoor quality through better ventilation and personal space, while ensuring fair reward structures. These holistic measures can mitigate stress, reduce negative behaviors, and foster a sustainable work environment. Future research should combine objective environmental data with longitudinal designs to further validate these findings.

## Supplementary Information

Below is the link to the electronic supplementary material.

Supplementary Material 1

## Data Availability

Data are available upon reasonable request.
