# Supplementary material for: Effects of indoor physical environment and psychosocial stress on work performance in a Thai academic setting
Source: Sci Rep. 2026 May 15;16:22189. doi: 10.1038/s41598-026-53266-0 (PMC13369939; doi:10.1038/s41598-026-53266-0)
Supplement: Supplementary file 1 — Supplementary Material 1 [file 41598_2026_53266_MOESM1_ESM.docx]

**Table 3:** The associated factors with individual work performance scores by hierarchical multiple linear regression analysis (n=419)

| **Variable** | **Task Performance** | | | | | **Contextual Performance** | | | | | **Counterproductive Work Behavior** | | | | |
| --- | --- | --- | --- | --- | --- | --- | --- | --- | --- | --- | --- | --- | --- | --- | --- |
|  | **Beta** | **95%CI** | | **p-value** | **Standardized beta** | **Beta** | **95%CI** | | **p-value** | **Standardized beta** | **Beta** | **95%CI** | | **p-value** | **Standardized beta** |
|  |  | **Lower** | **Upper** |  |  |  | **Lower** | **Upper** |  |  |  | **Lower** | **Upper** |  |  |
| **Model 1: demographics** | **Adjusted R² = 0.055** | | | | | **Adjusted R² = 0.003** | | | | | **Adjusted R² = 0.004** | | | | |
| **Gender** |  |  |  |  |  |  |  |  |  |  |  |  |  |  |  |
| Female | reference | - | - | - | - | reference | - | - | - | - | reference | - | - | - | - |
| Male | **-0.12*** | **-0.23** | **-0.01** | **0.03** | **-0.11** | -0.03 | -0.15 | 0.09 | 0.64 | -0.02 | -0.02 | -0.19 | 0.15 | 0.80 | -0.01 |
| **Age** | 0.00 | -0.01 | 0.01 | 0.47 | 0.08 | 0.00 | -0.01 | 0.01 | 0.89 | -0.01 | 0.01 | -0.01 | 0.03 | 0.24 | 0.13 |
| **Monthly Salary** |  |  |  |  |  |  |  |  |  |  |  |  |  |  |  |
| <15000 | reference | - | - | - | - | reference | - | - | - | - | reference | - | - | - | - |
| 15001-30000 | 0.01 | -0.15 | 0.18 | 0.87 | 0.01 | -0.17 | -0.36 | 0.03 | 0.10 | -0.12 | -0.09 | -0.36 | 0.17 | 0.48 | -0.05 |
| 30001-45000 | 0.05 | -0.19 | 0.28 | 0.70 | 0.03 | -0.18 | -0.45 | 0.10 | 0.22 | -0.09 | 0.01 | -0.37 | 0.38 | 0.98 | 0.00 |
| 45001-60000 | 0.29 | -0.11 | 0.69 | 0.16 | 0.08 | 0.11 | -0.36 | 0.58 | 0.65 | 0.03 | 0.33 | -0.30 | 0.96 | 0.30 | 0.06 |
| > 60000 | 0.23 | -0.08 | 0.54 | 0.15 | 0.10 | 0.11 | -0.25 | 0.47 | 0.54 | 0.04 | 0.20 | -0.29 | 0.68 | 0.42 | 0.05 |
| **Weekly Workhour** | 0.00 | 0.00 | 0.00 | 0.87 | 0.01 | 0.00 | 0.00 | 0.01 | 0.64 | 0.02 | 0.00 | -0.01 | 0.00 | 0.26 | -0.06 |
| **Job Duration** | 0.00 | -0.01 | 0.01 | 0.43 | 0.08 | 0.00 | -0.01 | 0.01 | 0.83 | 0.02 | -0.01 | -0.02 | 0.01 | 0.34 | -0.10 |
| **Model 2: IEQ** | **Adjusted R² = 0.003** | | | | | **Adjusted R² = 0.014** | | | | | **Adjusted R² = 0.011** | | | | |
| **Office Environment Satisfaction** |  |  |  |  |  |  |  |  |  |  |  |  |  |  |  |
| Temperature | 0.02 | -0.03 | 0.06 | 0.42 | 0.06 | -0.03 | -0.08 | 0.02 | 0.24 | -0.08 | 0.00 | -0.07 | 0.06 | 0.96 | 0.00 |
| Air movement | -0.01 | -0.05 | 0.03 | 0.73 | -0.02 | **-0.06*** | **-0.10** | **-0.01** | **0.01** | **-0.13** | 0.06 | -0.01 | 0.12 | 0.07 | 0.10 |
| Air Quality | 0.03 | -0.02 | 0.08 | 0.30 | 0.08 | 0.00 | -0.05 | 0.06 | 0.88 | 0.01 | -0.05 | -0.12 | 0.03 | 0.25 | -0.09 |
| Light | 0.01 | -0.03 | 0.05 | 0.63 | 0.03 | -0.01 | -0.06 | 0.04 | 0.59 | -0.03 | -0.01 | -0.08 | 0.06 | 0.77 | -0.02 |
| Noise | 0.00 | -0.04 | 0.04 | 0.99 | 0.00 | -0.04 | -0.08 | 0.01 | 0.15 | -0.09 | -0.04 | -0.10 | 0.03 | 0.25 | -0.07 |
| Privacy | 0.03 | -0.02 | 0.08 | 0.20 | 0.09 | **0.06*** | **0.00** | **0.11** | **0.04** | **0.15** | 0.03 | -0.04 | 0.10 | 0.43 | 0.06 |
| Layout | 0.03 | -0.03 | 0.10 | 0.32 | 0.10 | -0.03 | -0.11 | 0.04 | 0.41 | -0.08 | -0.04 | -0.15 | 0.06 | 0.41 | -0.08 |
| Decoration | -0.03 | -0.09 | 0.03 | 0.34 | -0.09 | 0.01 | -0.06 | 0.08 | 0.78 | 0.03 | 0.10 | 0.00 | 0.20 | 0.05 | 0.18 |
| **Model 3: ERI** | **Adjusted R² = 0.020** | | | | | **Adjusted R² = 0.001** | | | | | **Adjusted R² = 0.029** | | | | |
| **ERI ratio** |  |  |  |  |  |  |  |  |  |  |  |  |  |  |  |
| Low-risk (≤1) | reference | - | - | - | - | reference | - | - | - | - | reference | - | - | - | - |
| High-risk (>1) | **-0.17*** | **-0.28** | **-0.05** | **<0.01** | **-0.15** | -0.08 | -0.21 | 0.05 | 0.23 | -0.06 | **-0.31*** | **-0.48** | **-0.14** | **<0.01** | **-0.18** |

* *p-value* < 0.05
